# Role of the active cycle of breathing technique combined with phonophoresis for the treatment of patients with chronic obstructive pulmonary disease (COPD): study protocol for a preliminary randomized controlled trial

**DOI:** 10.1186/s13063-021-05184-x

**Published:** 2021-03-23

**Authors:** M. D. Shen, L. R. Guo, Y. W. Li, R. T. Gao, X. Sui, Z. Du, L. Q. Xu, H. Y. Shi, Y. Y. Ni, X. Zhang, Y. Pang, W. Zhang, T. Z. Yu, F. Li

**Affiliations:** School of Nursing, Jilin University, No 965, Xin Jiang Avenue, Changchun, 130000 Jilin Province China

**Keywords:** Airway cycle of breathing techniques, Phonophoresis, Chronic obstructive pulmonary disease, Protocol, Randomized controlled trial

## Abstract

**Background:**

Chronic obstructive pulmonary disease (COPD) is a chronic inflammatory lung disease characterized by coughing, the production of excess sputum, and dyspnea. Patients with excessively thick sputum may have frequent attacks or develop more serious disease. The guidelines recommend airway clearance for patients with excessive sputum who are hospitalized with COPD. The active cycle of breathing technique is the most common non-pharmacological airway clearance technique used by physiotherapists. However, the effectiveness of the technique is not always guaranteed. Active cycle of breathing techniques require the initial dilution of the sputum, usually by inhalation drugs, which may have limited effects. Recent studies have found that phonophoresis decreases inflammation, suggesting the potential of the combined usage of active cycle of breathing techniques and phonophoresis. Therefore, the aim of this study is to explore the effectiveness and safety of combining active cycle of breathing technique and phonophoresis in treating COPD patients.

**Methods and analysis:**

We propose a single-blind randomized controlled trial using 75 hospitalized patients diagnosed with COPD with excessive sputum production. The patients will be divided into three groups. The intervention group will receive active cycle of breathing techniques combined with phonophoresis. The two comparison groups will be treated with active cycle of breathing techniques and phonophoresis, respectively. The program will be implemented daily for 1 week. The primary outcomes will be changes in sputum viscosity and production, lung function, and pulse oximetry. Secondary outcomes include the assessment of COPD and anxiety, measured by the COPD Assessment Test scale and the Anxiety Inventory for Respiratory Disease, respectively; self-satisfaction; the degree of cooperation; and the length of hospital stay. All outcome measures, with the exception of sputum production and additional secondary outcomes, will be assessed at the commencement of the study and after 1 week’s intervention. Analysis of variance will be used to investigate differences between the groups, and a *p*-value of less than 0.05 (two-tailed) will be considered statistically significant.

**Discussion:**

This study introduces a combination of active cycle of breathing techniques and phonophoresis to explore the impact of these interventions on patients hospitalized with COPD. If this combined intervention is shown to be effective, it may prove to be a better treatment for patients with COPD.

**Trial registration:**

The trial was registered prospectively on the Chinese Clinical Trial Registry on 24 December 2019.ClinicalTrials.gov ChiCTR1900028506. Registered on December 2019.

## Article summary

Strengths and limitations of this study:

1. The use of phonophoresis in treating COPD patients is poorly documented.
2. The combination of active cycle of breathing techniques and phonophoresis is novel.
3. Study personnel are experienced physicians, rehabilitators, and caregivers, providing practical guidance for the research.
4. We propose a single-center randomized clinical trial. It is unclear whether our findings will be generalizable to other locations and clinical settings. However, the large geographic, cultural, and socioeconomic diversity of the inpatients may help to reduce this potential bias.

## Introduction

According to the statistics of the Global Burden of Disease Study, chronic obstructive pulmonary disease (COPD) has a high prevalence, as well as high mortality and morbidity [1–7]. In China alone, in 2015, nearly 100 million people were suffering from COPD [8]. Furthermore, the medical expenses of patients with COPD are increasing, resulting in a heavier economic burden. At present, COPD has become a global public health problem.

Cough, sputum production, and dyspnea are the main symptoms of COPD [9, 10]. Patients with excessively thick sputum may suffer frequent attacks or be prone to more serious disease [11]. To address the problem of excessive viscous sputum in patients hospitalized with COPD, the American Association for Respiratory Care (AARC) published the “AARC clinical practice guideline: effectiveness of non-pharmacologic airway clearance therapies in hospitalized patients.” This guideline [12] describes the necessity for airway clearance in such patients. A cross-sectional survey [13] in Sweden showed that 75% of physiotherapists prescribed airway clearance treatments for most COPD patients, and most physiotherapists (89%) believe that sputum clearance is an important aspect of the overall management of COPD patients. Of the various non-pharmacologic airway clearance techniques, active cycle of breathing techniques are the most commonly used by physiotherapists [14]. A systematic review [15] shows that the active cycle of breathing technique can improve lung function, levels of arterial blood gases, perceived levels of dyspnea, and quality of life. In addition, the systematic reviews of Cabillic et al. [16] and Ides et al. [17] observed that the active cycle of breathing technique was an effective treatment for patients with COPD, with a grade B level of evidence, in comparison to other airway clearance techniques.

Both expectoration and reducing lung inflammation are necessary in treating COPD. Phonophoresis is a physical therapy technique that combines ultrasound and topical medications [18]. It has been proven to decrease inflammatory infiltration [19] through the absorption of ultrasonic energy and cavitation [20] and has been widely studied as a non-invasive method of medication administration [21]. There is ample evidence showing that phonophoresis is both more effective and leads to greater patient compliance [22] than conventional methods for administering medication. Recent studies have demonstrated the efficacy of phonophoresis in the treatment of acute calcific tendinitis of the shoulder [23], chronic nonbacterial prostatitis [24], and chronic rhinosinusitis [25], among other diseases. At present, COPD patients in the Medical Center in Changchun, China, mostly use inhalation therapy to dilute the sputum. We propose exploring the use of phonophoresis combined with active cycle of breathing techniques to relieve the respiratory symptoms of COPD patients, which may be more effective than the use of a single treatment. To our knowledge, there are no reports of the use of this combined therapy for COPD. The aim of this single-blind, three-arm, randomized controlled trial, therefore, is to investigate combining active cycle of breathing techniques and phonophoresis in treating patients hospitalized with COPD.

## Methods

### Study design

This is a single-blind, three-arm, randomized controlled trial. The intervention group will receive active cycle of breathing techniques combined with phonophoresis and control group 1 will receive active cycle of breathing only, while control group 2 will receive phonophoresis only. By comparing these three groups, we hope to explore the benefits of a combined program, compared to a single intervention. For the three-armed trial [26], COPD patients will receive active cycle of breathing techniques during hospitalization. Because the mean length of hospital stay for COPD patients in our medical center is 1 week, the duration of the intervention in our study will be set at 1 week. The trial will be conducted at the Medical Center in Changchun, China. In line with the recommendations of the Protocol Items Recommendations for Interventional Trials (SPIRIT) guidelines [27], our research protocol has been registered with the China Clinical Trial Registration (ChiCTR1900028506) and has been approved by the Ethics Committee of Jilin University (2019122301). We expect to begin recruitment in December 2019 and end in June 2021. Any changes to the plan will be negotiated with trial participants and the ethics committee. A copy of the revised protocol will be sent to the patient involvement branch of the ethics committee to add to the Investigator Site File, and any deviations from the protocol will be fully documented using a breach report form.

### Eligibility and recruitment

Participants in this study are patients with COPD admitted to the Department of Respiratory Medicine, Affiliated Hospital of Jilin University. The inclusion and exclusion criteria are shown below.

Inclusion criteria:

- ① Hospitalized patients diagnosed with COPD according to the Global Initiative for Chronic Obstructive Lung Disease criteria
- ② Patients with clear consciousness, stable vital signs, and the ability to cooperate
- ③ Patients who have given informed consent
- ④ Adult patients
- ⑤ Patients with a sputum viscosity classified by the same person as grade II or grade III which is characterized by the loss of humidifying function in the upper respiratory tract, as well as the loss of the mucociliary transport function, and impaired expectoration [28]

Exclusion criteria:

- ① Presence of neuromuscular disease or terminal disease
- ② Presence of asthma or other respiratory diseases
- ③ History of thoracic and abdominal trauma and surgery
- ④ Presence of skin infection at the site of contact
- ⑤ Presence of a cardiac pacemaker, artificial stent, artificial valve, or heart failure
- ⑥ Presence of acute exacerbation of COPD or on a rehabilitation program

Patients meeting inclusion and exclusion criteria will be invited to participate in our research. Informed consent will be obtained from each participant. The recruitment process will be handled by an independent caregiver who is not involved in other procedures at the Medical Center in Changchun, China.

### Groups

#### Intervention group: active cycle of breathing techniques combined with phonophoresis

These patients will receive both active cycle of breathing techniques and phonophoresis during the 1-week intervention period. The active cycle of breathing technique consists of three main components [15]: breathing control, a thoracic expansion exercise, and a forced expiration technique.

##### Breathing control

Patients sit comfortably in a chair and breathe normally using the lower chest.

##### Thoracic expansion exercise

The physical therapist rests one hand on the epigastrium and guides the patients to breathe slowly and deeply using the lower chest. This is followed by holding the breath for 2 s and breathing out fully. This is repeated two or three times before returning to breathing control.

##### Forced expiration technique

The physical therapist guides the patient to inhale deeply, while simultaneously contracting the abdominal muscles and keeping the mouth and throat open. The patient then holds their breath for 2 s followed by vigorous exhalation, making a “ha” sound to stimulate coughing. The patient then returns to breathing control until ready to begin another cycle. Within 1 week of the intervention, each patient in this group will receive the active cycle breathing technique twice daily administered by the same physical therapist.

The phonophoresis procedures were developed by respiratory physicians, rehabilitators, caregivers, and other stakeholders. In this study, they will be led by the same experienced respiratory physician in the hospital who will not be involved in the active cycle of breathing technique. The physician checks the equipment and prepares a sterile patch before starting. Next, acetylcysteine is placed onto the sterile patch. Patients are auscultated and marked by the same experienced respiratory physician to determine the location of sputum accumulation. The emitter and the patch with the liquid are then applied in the marked position. The frequency will be adjusted to 20 kHz, with a depth of 10 mm–150 mm and intensity of 0.8 W/cm^2^, and treatment will continue for 20 min. This procedure is performed twice a day during the intervention period. If the patient has skin burns or intolerance during the execution, stop the intervention immediately.

#### Comparison group 1: active cycle of breathing technique

Comparison group 1 will receive the same active cycle of breathing, composed of breathing control, the thoracic expansion exercise, and forced expiration, as the intervention group. This is also performed twice daily during the 1-week intervention period, for a duration of 20 min per cycle. These patients will receive mucolytic agents by inhalation.

#### Comparison group 2: phonophoresis

The phonophoresis conductance instrument (NAVA-01TD) is used to administer the drug in the hospitalized COPD patients, using the same mucolytic agents that are used for the intervention group. The patients will undergo phonophoresis twice daily during the 1-week intervention period for 20 min each time.

### Outcome measurements

**Fig. 1:**
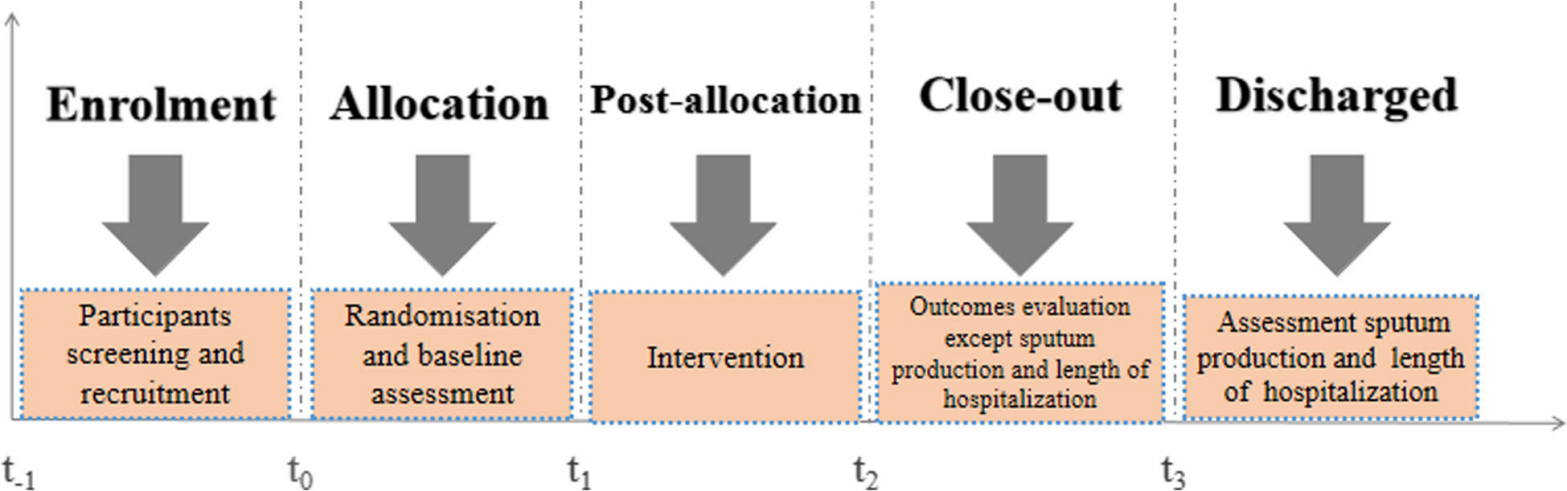
The profile of the outcome measures for the study

Outcome measures will be collected and analyzed by researchers blinded to the patients’ group allocation. All assessments will be performed at the commencement of the study and after the 1-week intervention, with the exception of assessments for sputum production and certain additional secondary outcomes (Fig. 1). Sputum production will be assessed after the 1-h intervention session and every 24 h during the week-long intervention period. Additional secondary outcomes will be collected only at the conclusion of the intervention. A summary of the outcome measures for the study is outlined in Table 1.

**Table 1:**
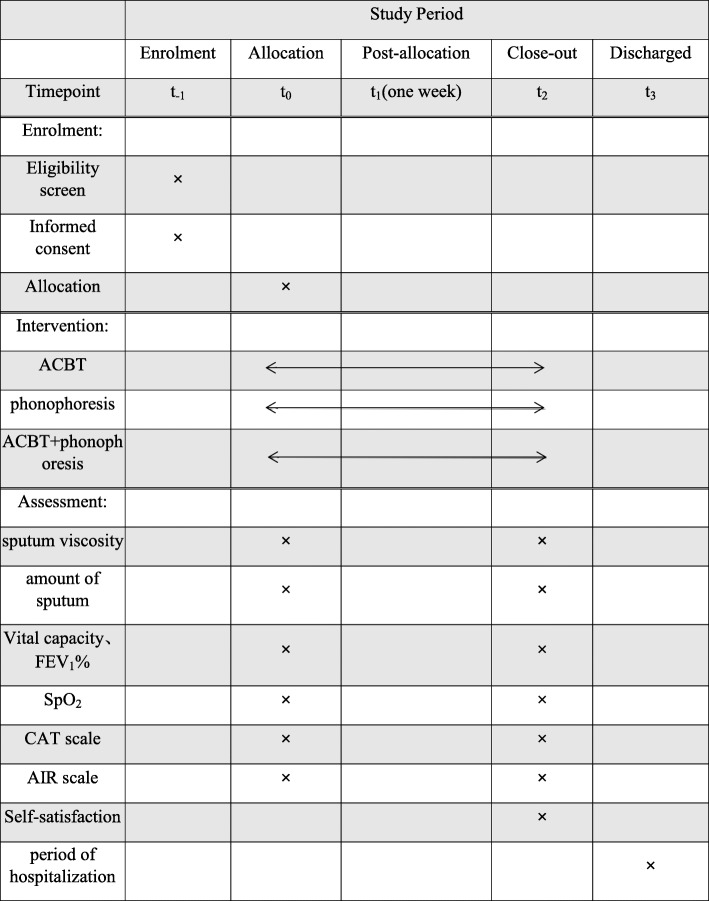
Time points of our research

*ACBT* active cycle of breathing technique, *FEV1%* forced expiratory volume in 1 s to the forced vital capacity, S*pO*_*2*_ arterial oxygen saturation, *CAT scale* COPD Assessment Test scale, *AIR scale* Anxiety Inventory for Respiratory Disease scale, *t*_*−1*_ enrolment, *t*_*0*_ baseline, *t*_*1*_ 1 week, *t*_*2*_ intervention close-out, *t*_*3*_ discharged

#### Primary outcomes

The first primary outcome will be sputum viscosity calculated using a viscometer [29, 30]. After the collection of sputum from patients, in line with the instructions, the assistant will insert the sputum into the rotor in the viscometer and read the digital output. The sputum viscosity is calculated by the formula (sputum viscosity = viscometer reading × 10). Patients will be given calibrated containers [31] at the baseline visit and instructed to collect 24 h worth of sputum in the container, between 07:00 each morning and 07:00 the following morning. The outcome assessor will observe and record the volumes of sputum at the 1- and 24-h time points daily during the intervention period.

We hope to reduce obstruction by clearing the sputum in the respiratory tract. Therefore, we will use the same pulmonary function meter to perform a complete spirometry assessment before and after the intervention [32]. Vital capacity can reflect the obstruction of pulmonary function and airway obstruction will be determined by FEV_1_%. The relation between expiration in the first second of forced expiration (FEV_1_) and the full forced vital capacity (FVC) will be represented by FEV_1_%, with normal values being approximately 75%. Generally speaking, the FEV_1_% of COPD patients is less than 70%. We are investigating whether this can be improved. The outcome assessor will only record vital capacity and FEV_1_% from the spirometry. We will follow the recommendations of the American Thoracic Society and European Respiratory Society [33] in the lung function test. Patients will be assessed between three and eight times, and the three best measurements with variability of less than 5% or 200 ml will be recorded [34]. The oxygen saturation of COPD patients will be expressed as SpO_2_. Similarly, SpO_2_ will be determined by pulse oximetry from the right middle finger [35–37] before and after the intervention. Since SpO_2_ is also a variable parameter, the patient will receive three evaluations and the average of the results recorded.

#### Secondary outcomes

The COPD Assessment Test scale (CAT scale) is a simple tool for assessing the health-related quality of life in COPD patients and is the recommended questionnaire in the global strategy for the diagnosis, management, and prevention of COPD [38]. Higher scores indicate a poorer outcome [39, 40]. The scale consists of eight points. Anxiety in patients with COPD will be assessed by the Anxiety Inventory for Respiratory Disease scale (AIR scale) [41]. The scale has ten points (score range, 0 [best]–30 [worst]), and a score of greater than eight indicates a high level of anxiety [42].

#### Additional secondary outcomes

Patient self-satisfaction, degree of cooperation, and the length of hospital stay will be additional secondary outcomes. Improvement in the patients’ condition, as well as their perception of the effectiveness of the interventions, will be evaluated by self-report on a scale of 0 to 100. After the intervention, the patients’ self-satisfaction will be scored on a 5-point Likert scale with 0 indicating “dissatisfied” and 4 meaning “very satisfied.” In addition, the proportion of patients who complete treatment during the study period will be used as the measure of their degree of cooperation [43]. These statistics will be compiled in the absence of the patient. The length of hospital stay will be measured in days [44].

### Sample size

Since the trial was designed with three arms, analysis of variance was chosen to calculate the sample size. We will have 90% power at the significance level (alpha) of 0.15 with an effect size of 0.4 to detect significant differences in sputum viscosity. Based on previous studies [45, 46], we assume that the dropout rate of our trial will be about 20% after the week-long intervention. Thus, the total sample size was 75, with 25 patients in each of the three groups. The sample size was calculated using the software PASS 2019.

### Randomization, blinding, and allocation concealment

**Fig. 2:**
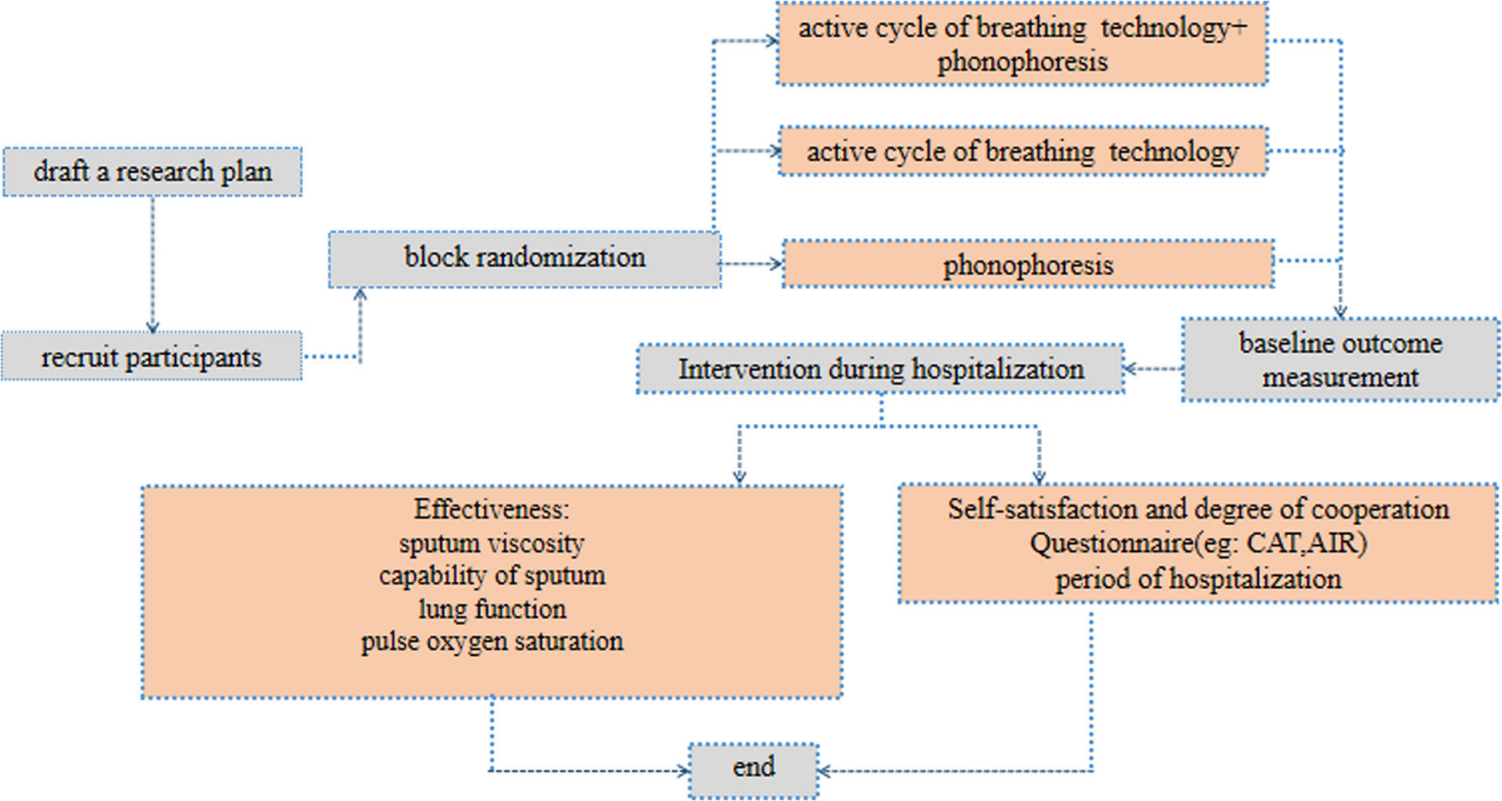
Profile of our research

We will screen 75 patients in strict accordance with the inclusion or exclusion criteria and then randomly divide them into three groups by computer-assisted block randomization stratified by disease severity. The random grouping will be performed by independent individuals who are not involved in the recruitment process. Sealed opaque envelopes will be used to conceal the patient distribution. Given the nature of the study, neither the intervention practitioner nor the participant can be blinded to the treatment plan, so blinding is only applicable for the data collection and evaluation. Neither patients nor the public will be involved in the design, conduct, reporting, or dissemination plans of our research. An illustration is shown in Fig. 2, and the time points are shown in Table 1.

### Data collection, management, and analysis

All patients’ data will be recorded by trained clinical researchers using a standardized case report form (CRF) and saved using the EpiData electronic database. To ensure the reliability of our study, the management of this trial will be conducted by the Trial Steering Committee (TSC). Main investigators and statisticians consist of our Trial Steering Committee, who will oversee the entire trial. Meetings will be held once a month to monitor the progress of the research and to share opinions when there are problems. The measurement data will be expressed by mean ± SD, and the count data will be expressed by frequency and percentage. A Kolmogorov-Smirnov test will be performed to analyze sample normality. Within-group changes in sputum viscosity, FEV_1_%, vital capacity, arterial oxygen saturation, quality of life, and the level of anxiety will be compared using a paired *t*-test or Wilcoxon test depending on the data distribution. Inter-group differences between baseline and post-intervention among the outcomes will be compared by one-way analysis of variance. According to the normality of the data distribution, a general linear model or multi-generalized linear model will be used to compare the differences between the primary and secondary outcomes after adjusting the covariates, including sociodemographic variables, such as sex, age, body mass index, smoking history, severity of disease, among others, and baseline variables. In addition, we will follow the principle of intention to treat (ITT) and use the sequence mean method to deal with the missing data to analyze the results of the comparison between groups of each outcome. To assess whether the missing data will affect the results of the study, we will compare participants who did not complete the intervention with those who completed the intervention to verify the sensitivity of the results of the trial. A two-tailed *p*-value of < 0.05 will be taken as significant. The data from this experiment will be entered by Epidata3.1 and analyzed by SPSS 26.0 software.

### Monitoring of adverse events

All adverse events that occur in our intervention will be monitored by researchers who are not involved in the data collection and have no conflicts of interest in terms of the data monitoring committee (DMC). Adverse events are defined as any untoward occurrences in study participants that are potentially related to the implementation of the study protocol. When a participant experiences an adverse event or a worsening of the disease, they will be immediately withdrawn from the trial, and statistical analysis will be performed with the existing data. The Ethics Committee will hold an annual meeting to monitor the execution of the entire trial.

### Patient and public involvement (PPI)

Patients and the public will not be involved in the processes of recruiting, randomizing, allocating, conducting interventions, and collecting outcomes. However, COPD patients who are not participants in the study may be involved in the test to discover other unintended effects of phonophoresis before the start of the trial. In addition, to avoid adding to the research burden, the outcomes of the study were selected in line with the priorities and preferences of patients.

### Ethics and dissemination

There is no anticipated harm and will be no compensation for trial participation. Research reports will be disseminated through scientific forums, including peer-reviewed publications and presentations at national and international conferences. The datasets analyzed during the current study will be available from the corresponding author on reasonable request.

## Discussion

The study will explore the efficacy of a program combining the active cycle of breathing technique and phonophoresis in patients with COPD. The purpose of our study is to investigate whether the combined treatment can improve sputum viscosity, expectoration, and lung function.

Over-production and increased viscosity of sputum is common among COPD patients. Previous studies have attempted to solve these problems with active cycle of breathing techniques, without entirely satisfactory results [15–17]. It can be seen from previous reports that airway clearance is a complex process, and adequate expectoration may take a long time to resolve satisfactorily [47]. We thus propose an intervention program of active cycle of breathing techniques combined with phonophoresis to explore the best way for sputum management among COPD patients. Airway clearance techniques [48], medication [49], and rehabilitation exercise training [50] are effective in the removal of sputum. Researchers have, therefore, combined exercise training with physiotherapy applied to young patients with cystic fibrosis, a comprehensive intervention project that has resulted in significant improvements on sputum production, oxygen saturation, and short-term lung function [48]. The application of active cycle of breathing techniques combined with phonophoresis in COPD patients is not well documented. Phonophoresis can markedly enhance the effectiveness of drugs in the acute exacerbation of inflammation and pain and has received increasing attention recently [51–55]. We hope that the outcomes will be optimized by promoting more effective absorption of the drugs and the comprehensive program can play a complementary role in the treatment of COPD patients.

Due to the high incidence of COPD [8], it is likely that participants for this study will be relatively easy to recruit. In addition, the establishment of a pulmonary rehabilitation training center can provide practical guidance for the implementation of interventions. Previous research results are also able to provide us with a theoretical basis. Svenningsen et al. [45] reported a cross-over control trial of COPD airway clearance in which the loss on follow-up in the intervention and control groups was 12.50% and 18.75%, respectively, while Nicolini et al. [46] found that the loss on follow-up of the intervention groups 1 and 2 and the control group were 12.50%, 10%, and 17.50%, respectively. However, the dropout rate is unlikely to be high in our study, as it is conducted in the hospital where the rate of loss of study participants is certainly lower than that outside the hospital.

There are limitations to our research. Firstly, this is a single-center randomized clinical trial, and it is unclear whether our findings are generalizable to other locations and clinical settings. Secondly, as the study is of relatively short duration, no data will be available beyond 1 week and longer-term effectiveness will not be able to be evaluated.

In summary, this study introduces a combination of active cycle of breathing techniques and phonophoresis to explore the impact on patients hospitalized with COPD. If this comprehensive intervention is shown to be effective, it may prove to be a better treatment for patients with COPD.

### Trial status

When the manuscript is completed, the trial is in the recruitment stage. Our recruitment started in December 2019. This protocol is version 1.0, dated 25 December 2019. The end of the trial is expected in June 2021.

## Data Availability

The clinical data collected will not be shared with the public. However, non-clinical data will be shared with the public and other researchers.

## Abbreviations

COPD: Chronic obstructive pulmonary disease
CAT: Chronic obstructive pulmonary disease assessment test scale
AIR: Anxiety Inventory for Respiratory Disease Scale
AARC: American Association for Respiratory Care
GOLD: Global Initiative for Chronic Obstructive Lung Disease
ACBT: Active cycle of breathing technique
PPI: Patient and public involvement
FEV1%: Forced expiratory volume in 1 s to forced vital capacity
SpO_2_: Pulse oxygen saturation
